# Factors influencing U.S. canine heartworm (*Dirofilaria immitis*) prevalence

**DOI:** 10.1186/1756-3305-7-264

**Published:** 2014-06-06

**Authors:** Dongmei Wang, Dwight D Bowman, Heidi E Brown, Laura C Harrington, Phillip E Kaufman, Tanja McKay, Charles Thomas Nelson, Julia L Sharp, Robert Lund

**Affiliations:** 1 Department of Mathematical Sciences, Clemson University, Clemson, SC 29634-0975, USA; 2 Department of Microbiology and Immunology, College of Veterinary Medicine, Cornell University, Ithaca, NY 14853, USA; 3 Division of Epidemiology and Biostatistics, College of Public Health, University of Arizona, Tucson, AZ 85724, USA; 4 Department of Entomology, Cornell University, Ithaca, NY 14853, USA; 5 Entomology and Nematology Department, University of Florida, Gainesville, FL 32611, USA; 6 Department of Biological Sciences, Arkansas State University, State University, AR 72467, USA; 7 Animal Medical Center, Anniston, AL 36201, USA

**Keywords:** Canine heartworm, *Dirofilaria immitis*, Head-banging smoothing, Mosquito vectors, Prevalence rates

## Abstract

**Background:**

This paper examines the individual factors that influence prevalence rates of canine heartworm in the contiguous United States. A data set provided by the Companion Animal Parasite Council, which contains county-by-county results of over nine million heartworm tests conducted during 2011 and 2012, is analyzed for predictive structure. The goal is to identify the factors that are important in predicting high canine heartworm prevalence rates.

**Methods:**

The factors considered in this study are those envisioned to impact whether a dog is likely to have heartworm. The factors include climate conditions (annual temperature, precipitation, and relative humidity), socio-economic conditions (population density, household income), local topography (surface water and forestation coverage, elevation), and vector presence (several mosquito species). A baseline heartworm prevalence map is constructed using estimated proportions of positive tests in each county of the United States. A smoothing algorithm is employed to remove localized small-scale variation and highlight large-scale structures of the prevalence rates. Logistic regression is used to identify significant factors for predicting heartworm prevalence.

**Results:**

All of the examined factors have power in predicting heartworm prevalence, including median household income, annual temperature, county elevation, and presence of the mosquitoes *Aedes trivittatus*, *Aedes sierrensis* and *Culex quinquefasciatus*. Interactions among factors also exist.

**Conclusions:**

The factors identified are significant in predicting heartworm prevalence. The factor list is likely incomplete due to data deficiencies. For example, coyotes and feral dogs are known reservoirs of heartworm infection. Unfortunately, no complete data of their populations were available. The regression model considered is currently being explored to forecast future values of heartworm prevalence.

## Background

The Companion Animal Parasite Council (CAPC) has compiled a data set of over nine million heartworm antigen tests performed on dogs in the United States during 2011 and 2012 [1]. These data are test results taken from dogs that visited veterinary clinics throughout the United States. From this data, our goal is to quantify the environmental, socio-economic, and vector factors that influence canine heartworm prevalence rates. Brown *et al.*[2] describe the data and list factors posited to influence canine heartworm prevalence rates (these are discussed fully below). This paper quantitatively assesses which of the factors are important (or unimportant) and in what direction the factors impact (increase or decrease) heartworm prevalence rates.

The data will be used to estimate the probability that a dog entering a veterinary clinic will test positive for heartworm. Although prevalence rates increase if a dog is from an area with a high heartworm transmission rate, the raw data do not describe transmission, but rather the risk that a dog’s infection is detected if it enters a clinic within the United States and is tested for heartworm for any reason whatsoever. The most common reasons for testing a dog are: assessing the negative status of a dog before it begins heartworm prevention, annual testing of dogs on preventive prophylaxis to verify that the dog has been protected, and assessing whether or not a dog with clinical signs suggestive of heartworm disease is indeed infected. In many areas of the United States, dogs are kept on prophylaxis year round; however, in some areas, veterinarians utilize heartworm prevention seasonally. Here, annual testing verifies whether or not infection occurred during the period when preventives were not taken. To assess true transmission rates, it would be more appropriate to follow dogs or other canines, specifically coyotes, that are not receiving any form of prophylaxis [3,4]— these are not the canines studied here. According to the unpublished abstract of Pulaski *et al*. for the 58th Annual Meeting of the American Association of Veterinary Parasitologists and the unpublished presentation of Blagburn *et al.* at The Triennial Symposium of the American Heartworm Society in 2013, in parts of the United States, resistance to heartworm preventatives has been recognized. Thus, future data may help detect preventive failure.

A spatial logistic regression model will be fitted to the CAPC county-by-county heartworm test results and related to factor measurements. Logistic regression methods are used in lieu of ordinary regression techniques because prevalence probabilities, which must lie in the interval [0,1], are being modeled. A significant technical challenge involves the large number of counties reporting a small number of tests (often this count is zero). Small sample sizes from isolated counties can adversely impact results if not properly handled. Therefore, methods are developed that account for sample size issues. The head-banging algorithm, a method for smoothing the county-by-county prevalence rates, will be used to extract general spatial structure in the prevalence estimates; this procedure is adept at dealing with outlying observations and boundary (edge) features.

Our results are useful in a variety of contexts. First and foremost, predicting heartworm prevalence rates alerts the pet owner to high-risk areas. This will be evident from the baseline risk maps constructed in Section "Construction of the baseline heartworm prevalence map". Second, pinpointing the factors accompanying high heartworm prevalence rates provides an opportunity to target those factors in mosquito and heartworm control programs. Third, our results provide a quantitative analysis of canine heartworm across the entire country, allowing us to confirm that cases do occur in the Western United States. Finally, the fitted regression model can be used to forecast future prevalence levels of heartworm or its response to climate change.

## Methods

### The data and factors

This section describes the data provided by CAPC and lists the factors considered by our analysis.

**Figure 1:**
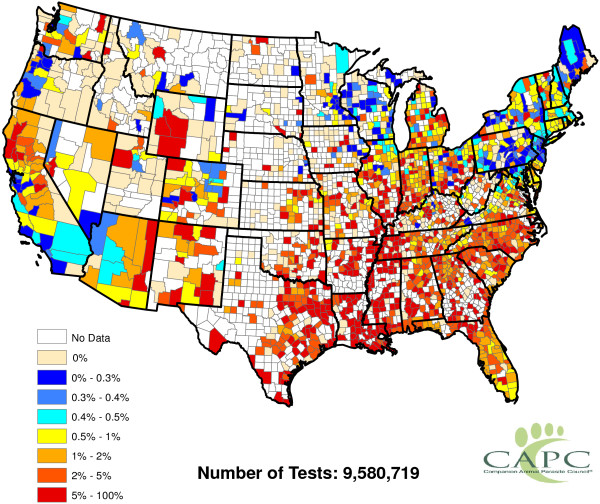
Raw reported heartworm prevalence rates for 2011 and 2012.

**Figure 2:**
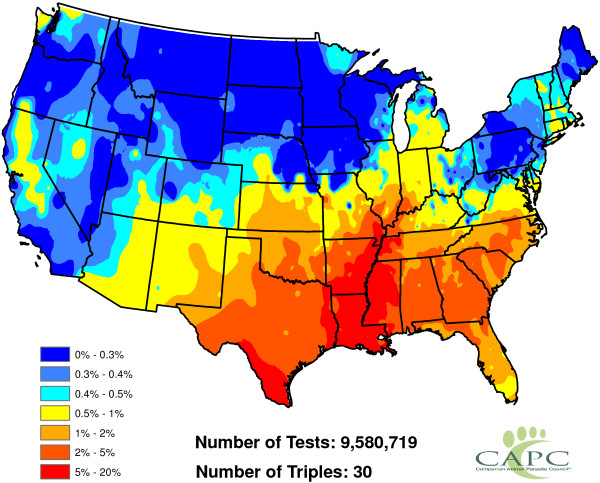
Head-banging smoothed heartworm prevalence rates for 2011 and 2012.

The raw test results were supplied by the Antech and IDEXX corporations [5,6] and report whether each performed test was positive or negative — no uncertainty margin is supplied for the results. While individual tests are reported by zip-code of the testing clinic, the raw data were aggregated into the number of positive tests and the total number of tests conducted over each calendar year in each county of the conterminous United States. Since only two calendar years of records are available, no attempt is made to include seasonal structure. The IDEXX samples represent both the results of pet-side heartworm antigen test kit and results from an IDEXX capture system [Heartworm RT and the 4Dx Plus (and originally 4Dx tests)] along with tests run by the IDEXX diagnostic laboratories [Heartworm Antigen by ELISA-Canine*]. Antech tests were performed at Antech Laboratories and utilized the Dirochek Assay and the AccuPlex4 heartworm antigen detection assay. Over 2011 and 2012, there were 9,580,719 total tests performed by either method, of which 111,259 were positive. Rudimentary statistical checks do not show vast differences between Antech and IDEXX samples.In most of the southeastern United States, veterinarians assume that outdoor dogs are at risk of heartworm infection, and thus, recommend that clients place their dogs on preventive protection. This may not be practical for all pet owners due to costs. In the CAPC data, many counties in the eastern United States report a small number (say less than 20) of tests. This is likely because tests are not being reported or are incommensurate with CAPC protocols, not because they are uncommonly performed. Tests from such counties are likely performed for the same reasons as other southeastern counties reporting a greater number of tests. In other areas of the United States, such as Montana or Idaho, testing is likely only performed if dogs have signs suggestive of heartworm disease and the veterinarian requires a confirmative test. Sometimes, veterinarians are aware that dogs travel to heartworm endemic areas for part of the year. These dogs may be tested annually when they return to their home state. Also, as is evident from Figures 1 and 2, it is now fairly obvious that heartworm occurs in much of the Western United States. In some of these areas, heartworm testing is probably conducted for the same reasons as more prevalent areas. Overall, while it is understood that there may be some sampling biases in certain areas of the United States, the CAPC data seems fairly reflective of a true random sample for many counties in the United States.

Other data aspects are worth illuminating. First, heartworm infections are not detectable by almost all testing methods until about 6 or 7 months after the dogs have become infected; this is how long it takes for either microfilariae or antigen to appear in the blood after infection. Thus, many of the detected infections likely commenced the year prior to a positive test result. Because no travel information exists, it cannot be known where a dog acquires infection. However, it is suspected that the majority of infected dogs were infected close to home. Second, it is not known if a dog has been tested more than once. Dogs may be tested more than once annually to verify the need for treatment, to verify successful treatment, or annually tested after the infection is first identified.

**Figure 3:**
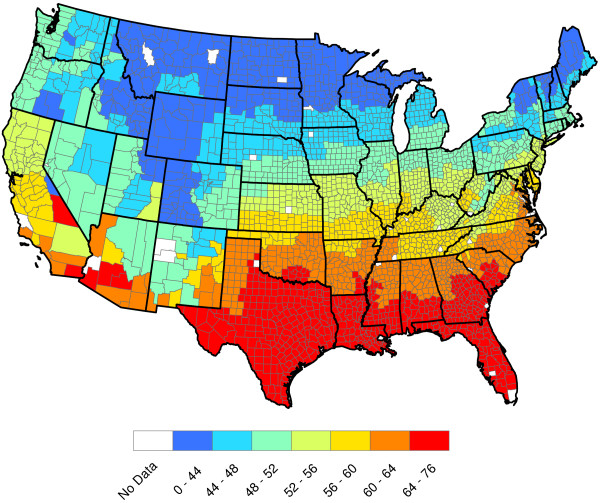
**2011 U.S. annual average temperature (Degrees F).** The temperature data included in this study were annual in nature and were aggregated by the National Climatic Data Center (NCDC) [7] by climate region [8]. These data are not county-by-county — all counties within a climate region are assigned the same annual temperature. For example, the state of Alabama has 67 counties and 8 climate regions. Annual temperatures for 2011 were used to generate this graphic. Temperature dependence on latitude is clear.

**Figure 4:**
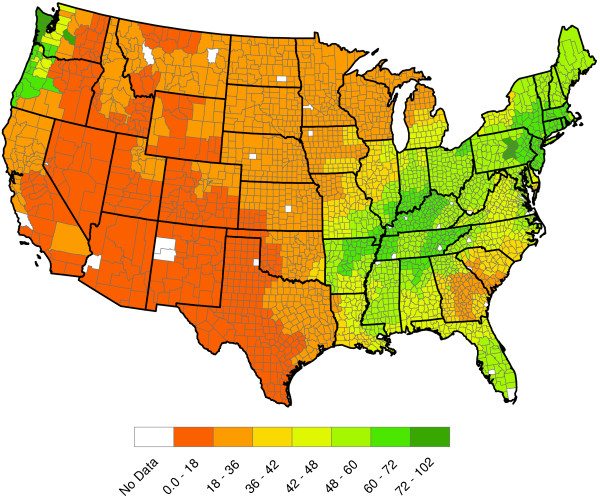
**2011 U.S. annual total precipitation (Inches).** The precipitation data were also obtained from the NCDC and has the same spatial resolution as the temperature data. Data used in this figure are for 2011. One sees a relatively dry Southwestern United States and higher precipitation in the southeastern United States.

**Figure 5:**
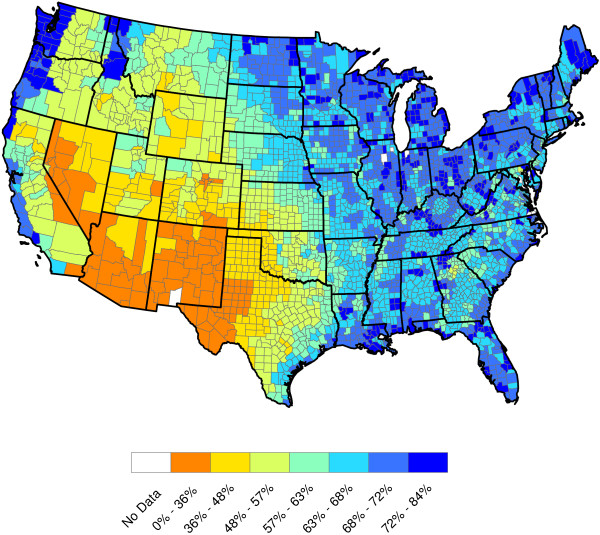
**2011 U.S. annual relative humidities (Percent).** Our humidity data were relative (and not absolute) humidities. Relative humidity is not measured directly, but can be estimated from temperature and dew point (dew point is measured) via $\text{RH}=\frac{\exp\left(\frac{17.27(D-32)5/9}{(D-32)5/9+237.3}\right)}{\exp\left(\frac{17.27(T-32)5/9}{(T-32)5/9+237.3}\right)}100\%,$ where *T* is annual temperature, *D* is annual dew point, and exp is the exponential function [9]. As expected, the Southeast is the most humid region in the United States.

**Figure 6:**
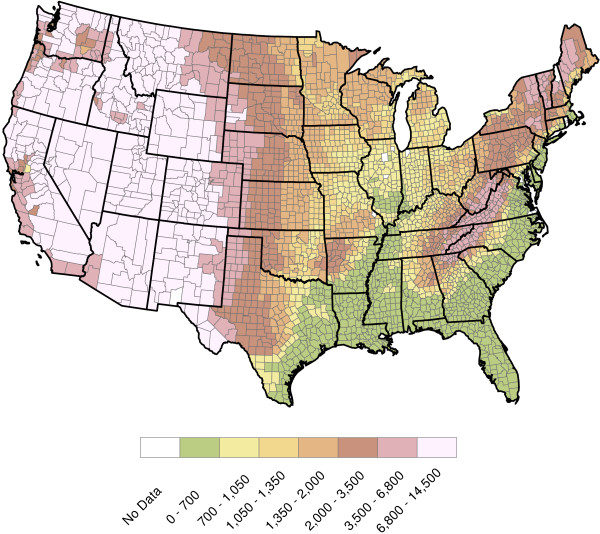
**U.S. county elevations (Feet).** Elevation data were obtained on a county-by-county [10] basis, with height of the highest point in each county used to produce this figure. Data containing average county elevations would be preferable to use but were not readily available. Of course, any biases should be minor in the eastern United States where most counties are homogeneous in elevation. However, the western States are less homogeneous. For example, Inyo County in California contains both the highest (Mount Whitney, 14,505 ft.) and lowest points (Death Valley, -282 ft.) in the conterminous United States. These limitations aside, elevation is a potentially important factor for heartworm prevalence as higher elevations are often associated with drier conditions.

**Figure 7:**
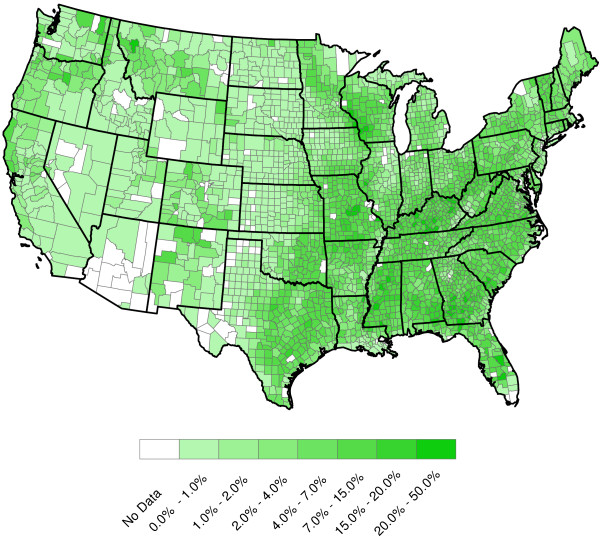
**2007 U.S. county forestion coverage (Percent).** County forest coverage was obtained from the United States Department of Agriculture (USDA) [11]. Total county area was obtained from the Census Bureau [12]. Percentages of forest coverage were calculated by dividing the forest coverage area by county area (times 100%). The definition of forest coverage here is restricted to agriculture woodland, which means land supporting trees capable of producing timber or other wood products, including but not limited to logs, lumber, posts, and firewood. This data are updated by the USDA every five years. However, the 2012 data is not available yet; hence, the 2007 data were used to generate the graph.

**Figure 8:**
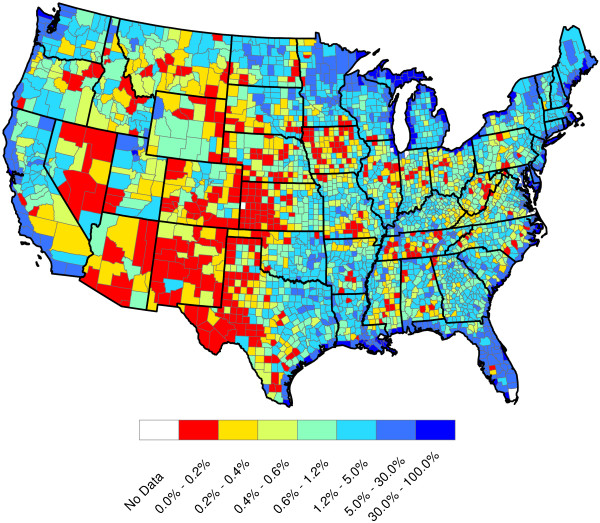
**2007 U.S. county water coverage (Percent).** County surface water coverage was obtained from the Census Bureau [12] and was calculated by dividing the surface water area by total county area reported in the Census Bureau [12]. The surface water coverage data were last updated in 2011, which were used in our analysis.

**Figure 9:**
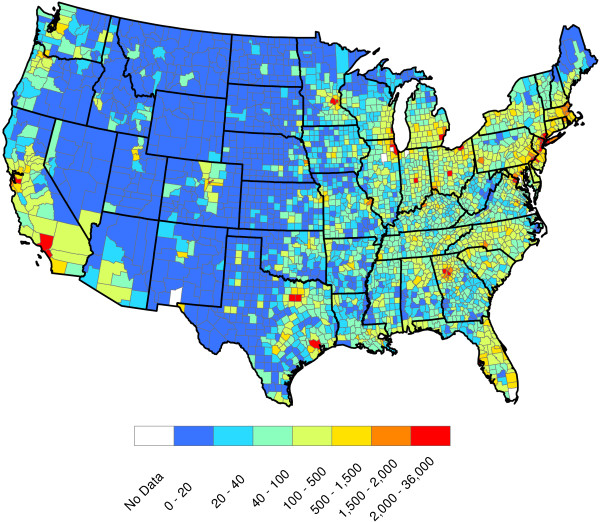
**2010 U.S. population density (100 people per square mile).** Population densities were calculated by dividing the number of 100 people in each county by the county area. The county populations and areas were taken from the most recent (2010) census data (Census Bureau [13]).

**Figure 10:**
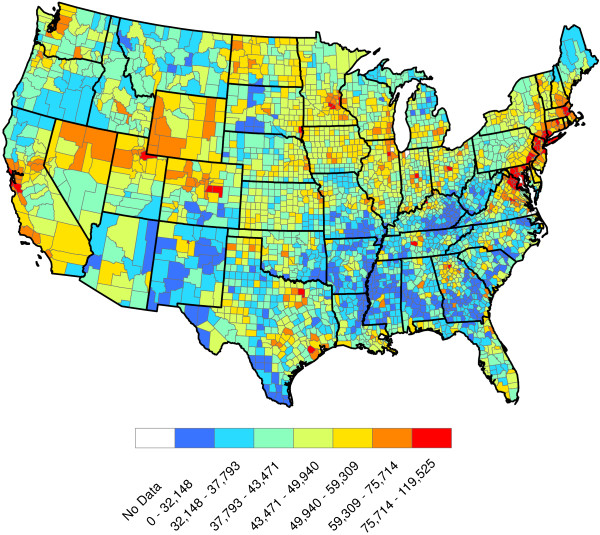
**2011 U.S. median household income (Dollars).** Median household incomes were obtained from the Census Bureau (2010 census). These data were adjusted for inflation based on 2010 dollars; the Census Bureau adjusts by multiplying 2011 median household income by the ratio of the Consumer Price Index of 2010 and 2011 [14].

**Figure 11:**
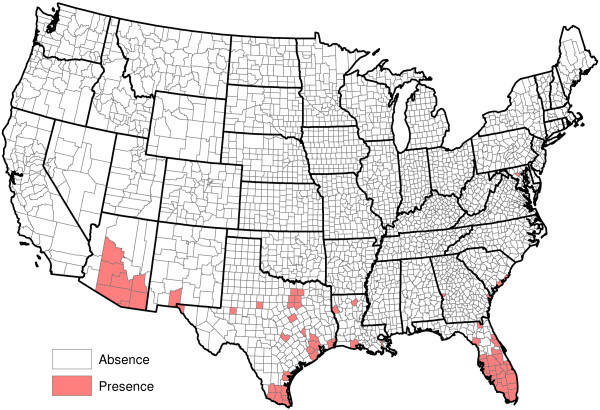
**Presence of** ***Aedes aegypti*.**

**Figure 12:**
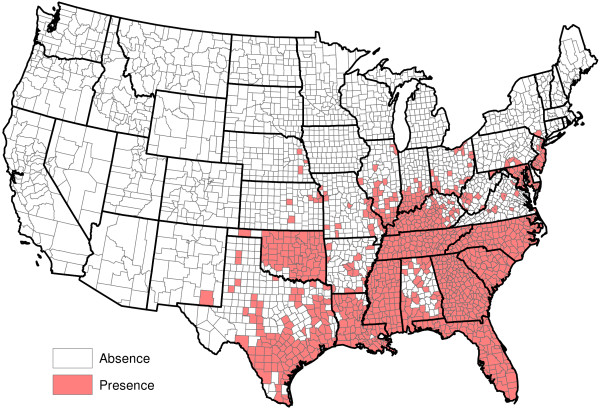
**Presence of** ***Aedes albopictus*.**

**Figure 13:**
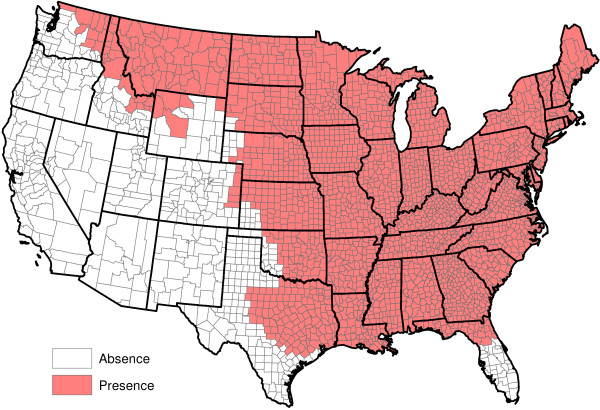
**Presence of** ***Aedes canadensis*.**

**Figure 14:**
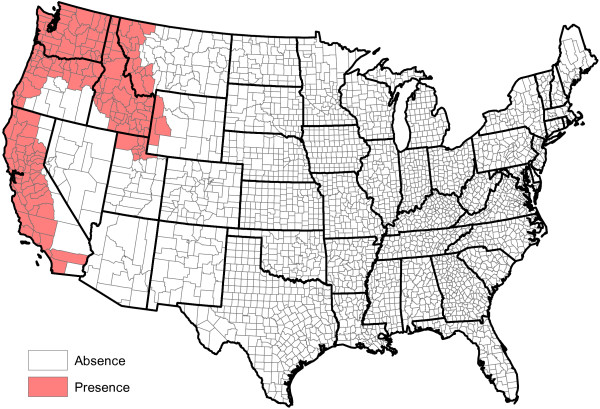
**Presence of** ***Aedes sierrensis*.**

**Figure 15:**
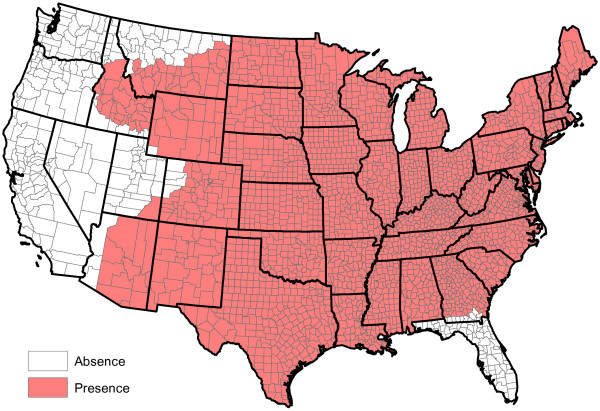
**Presence of** ***Aedes trivittatus*.**

**Figure 16:**
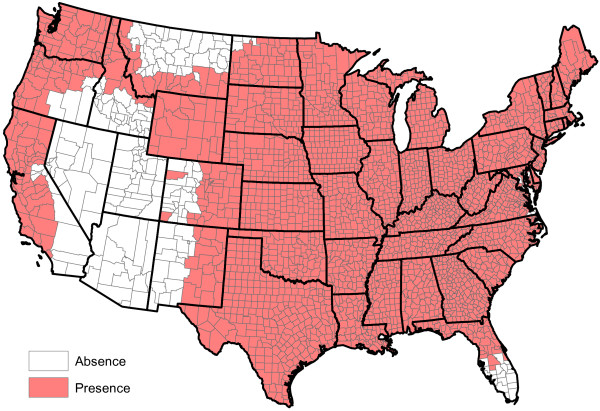
**Presence of** ***Anopheles punctipennis*.**

**Figure 17:**
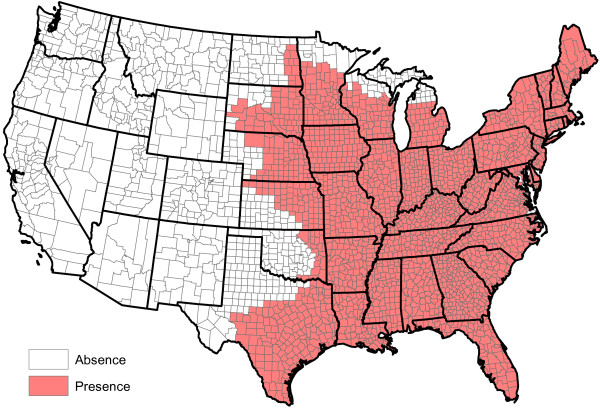
**Presence of** ***Anopheles quadrimaculatus*.**

**Figure 18:**
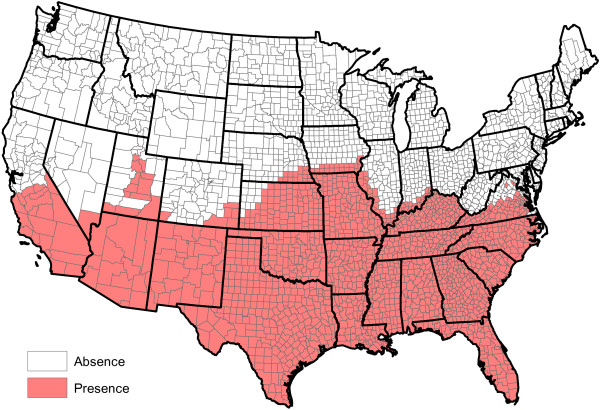
**Presence of *Culex quinquefasciatus *.**

The factors chosen for inclusion in this study are those envisioned to impact whether a dog is likely to have heartworm. These factors are a subset of those listed in Brown *et al.*[2] and contain climate variables (annual temperature, precipitation, and relative humidity; Figures 3, 4, 5); geographic factors (elevation, forest coverage, surface water coverage; Figures 6, 7, 8); societal factors (human population density and household income; Figures 9 and 10); and the presence or absence of *Aedes aegypti*, *Aedes albopictus* and six other mosquito species (Figures 11, 12, 13, 14, 15, 16, 17, 18). Presence or absence of mosquito species was used because abundance data are not available. Table 1 lists all considered factors. Many (but not all) of the factors are available on a county-by-county basis across the United States. Our methods do not *a priori* assume that all factors significantly influence heartworm prevalence rates, but rather seek to determine which factors significantly influence prevalence.

**Table 1:** Heartworm factors considered for inclusion in the study

	**Factors**	**Data available period**	**Scale**	**Source**
Climate factors	Annual temperature	2011 and 2012	Division	National Climate Data Center (NCDC)
	Annual precipitation	2011 and 2012	Division	NCDC
	Annual relative humidity	2011 and 2012	Station	NCDC
Geographic factors	Elevation	2012	County	http://www.cohp.org/
	Percentage forest coverage	2007	County	United States Department of Agriculture (USDA)
	Percentage surface water coverage	2010	County	U.S. Census Bureau
Societal factors	Population density	2010	County	U.S. Census Bureau
	Median household income	2011	County	U.S. Census Bureau
Mosquito species	*Aedes aegypti*	2008	County	Moore, CG. [15]
	*Aedes albopictus*	2012	County	Hynes NA [16]
	*Aedes canadensis*	2004	County	RF Darsie, Jr. and RA Ward [17]
	*Aedes sierrensis*	2004	County	RF Darsie, Jr. and RA Ward
	*Aedes trivittatus*	2004	County	RF Darsie, Jr. and RA Ward
	*Anopheles punctipennis*	2004	County	RF Darsie, Jr. and RA Ward
	*Anopheles quadrimaculatus*	2004	County	RF Darsie, Jr. and RA Ward
	*Culex quinquefasciatus*	2004	County	RF Darsie, Jr. and RA Ward

### Construction of the baseline heartworm prevalence map

We first construct a baseline heartworm prevalence map. This is done on an annual basis as there is too little data to consider seasonal effects. As will become apparent, this analysis is a necessary precursor to assess factor importance — informative factors should be able to reproduce the structure of our baseline prevalence map. For the years 2011 and 2012, all data were combined into a single sample.

For a county *s*, let *p*(*s*) denote the probability that a single dog tests heartworm positive. For notation, *n*(*s*) is the number of tests in county *s* and *k*(*s*) is the number of positive tests at county *s*. For example, if county *s* has 3 positive tests out of 100 during 2011 and eight positive tests out of 200 during 2012, then *k*(*s*) = 11, *n*(*s*) = 300, and $\hat{p}(s)=11/300$ (a hat over a quantity indicates it is an estimate). Figure 1 displays county-by-county values of $\hat{p}(s)$. This figure indicates that heartworm is most problematic in the Lower Mississippi Valley. The role of factors in explaining the prevalence rates will be discussed in Section "Factor quantification". No factors are involved in the calculation of $\hat{p}(s)$.

Since the number of dogs tested in distinct counties greatly varies, the raw values of $\hat{p}(s)$ need to be weighted. Estimated values of *p*(*s*) are more accurate for a sample of 100 dogs than for a sample of 10 dogs. To quantify this, the classical standard error is used. In particular, the estimated variance of $\hat{p}(s)$ is

(1) $$\hat{\text{Var}(\hat{p}(s))}=\frac{\hat{p}(s)(1-\hat{p}(s))}{n(s)}.$$

The estimated standard error of $\hat{p}(s)$ is the square root of (1). We weight the values of $\hat{p}(s)$ inversely proportional to this standard error. Before doing this, an adjustment was made to the values of $\hat{p}(s)$ for small sample sizes.

Technically, counties where all tests are positive or negative have $\text{Var}\left(\hat{p}(s)\right)=0$ (hence, its reciprocal is infinite), which could adversely impact our ensuing smoothing methods. To combat this, the Wilson estimator that adds two to numerator counts and four to denominator counts is used in lieu of $\hat{p}(s)$:

$${\hat{p}}_{W}(s)=\frac{k(s)+2}{n(s)+4}.$$

This estimator has desirable sampling properties and cannot be zero or unity [18]. Of course, for large *k*(*s*) and *n*(*s*), $\hat{p}(s)$ and ${\hat{p}}_{W}(s)$ are approximately equivalent.

The raw values of ${\hat{p}}_{W}(s)$ greatly vary across counties. Even counties in close proximity to one another often have highly different prevalence estimates. This said, some spatial structure clearly exists in Figure 1. Our next goal is to extract and explore this structure. To accomplish this, the weighted head-banging spatial smoothing algorithm was applied to the county-by-county values of ${\hat{p}}_{W}(s)$. This procedure serves to remove localized small-scale variations due to random chance, illuminating large-scale structures that are actual features of the prevalence rates.

While we will not delve into the details of weighted head-banging procedures, the technique is a median-based algorithm proposed by Tukey and Tukey [19] for smoothing spatial data. One needs to input the longitude and latitude of the centroid of each county, the county value to be smoothed, and the corresponding weights. Mungiole *et al.*[20] discuss the algorithm in detail. Head-banging takes its name from a child’s game, where the child presses their face against pins protruding from a board that are of various lengths. The result leaves an impression of the child’s face, smoothing the lengths of adjacent nails but leaving the general structure of the face’s impression. Head-banging techniques are very effective for down-weighting or removing noisy ‘spikes’ while preserving edge structures. A spike is an isolated observation that lacks confirmation from nearby data. Because of different testing practices from county to county, many spikes exist in the heartworm prevalence estimates. An edge occurs where data changes significantly in pattern — perhaps due to a mountain range. Edges are informative as they often demarcate distinct data regions.

To run the weighted head-banging algorithm, a parameter called the number of triples must be selected and the weights need to be specified. At each county where data is present, a set of triples (a triple for a county is represented by the county itself and two nearby counties) were selected based on the criteria proposed by Hansen [21]. The weight of county *s*, denoted by *w*(*s*), comes from the inverse of the standard error of $\hat{p}(s)$, with ${\hat{p}}_{W}(s)$ replacing $\hat{p}(s)$:

$$w(s)=\sqrt{\frac{n(s)}{\frac{k(s)+2}{n(s)+4}\left(1-\frac{k(s)+2}{n(s)+4}\right)}}.$$

Figure 2 shows our smoothed prevalence rates based on the weighted head-banging procedure. The larger the triple parameter is, the smoother (less rough) the resulting map will be. We have intentionally left the graphic slightly under-smoothed. This is because it is easy to visually smooth variabilities away with the eye, but impossible to recover true fluctuations that are erroneously smoothed away. Thirty triples were used to produce this graphic.

Figure 2 has interesting implications. First and foremost, heartworm is most prevalent in the Lower Mississippi Valley. While the northern latitudes show less activity, places where the prevalence rates were relatively higher do exist. Michigan, Vermont, and Northwest Washington, for example, show greater heartworm disease prevalence than some of the other states at the same latitude. The Northern Rockies perhaps show the least heartworm disease prevalence. While many inferences can be made from Figure 2, we caution the reader not to over-interpret minutia. The map is constructed from only two years of data and there are variations in the results that may be spurious. For example, two very close locations — say Baton Rouge and New Orleans, Louisiana — might be shaded different colors on the map, but should not be expected to have radically different prevalence rates. As additional years of data are collected, we expect our baseline to become more accurate. Another issue involves dispersal of the disease: there is no *a priori* reason to think that prevalence rates are static in time. With only two years of observations, time trends will be difficult to discern and are not explored herein.

### Factor quantification

This section examines the significance of the individual factors presented in Table 1 in predicting heartworm prevalence. A logistic regression model was created using data from 2011 and 2012. Logistic regression methods (as opposed to ordinary regression methods) [22] are specifically designed for cases involving a binary outcome that can be summarized by a probability, and hence limited to take values in the interval [0,1]. Our goal is to reproduce the structure in Figure 2.

Let *X*(*s*) = (*f*_1_(*s*),…,*f*_8_(*s*);1_1_(*s*),…,1_8_(*s*))^′^ be the collection of all predictive factors at county *s*. The logistic regression model attempts to explain spatial variations in *p*(*s*) from the factors via

(2) $$p(s)=\frac{e^{g(X(s))}}{1+e^{g(X(s))}},$$

where *g*(*X*(*s*)) has form

(3) $$\begin{array}{ll} \;g(X(s)) & =\text{logit}(p(s))=\ln\left(\frac{p(s)}{1-p(s)}\right)\\ & =\beta_{0}+\overset{8}{\underset{i=1}{\sum }}\beta_{i}f_{i}(s)+\overset{8}{\underset{i=1}{\sum }}\gamma_{i}1_{i}(s). \end{array}$$

Clarifying terms, ln denotes natural logarithm and logit(*x*) = ln(*x*)- ln(1 - *x*). Notice that *e*^*g*(*X*(*s*))^/(1 + *e*^*g*(*X*(*s*))^) ∈ [0,1] for any value of *g*(*X*(*s*)). This guarantees that all predicted prevalence rates lie between zero and unity. The overall location parameter, *β*_0_, is common to all counties while *β*_1_,…,*β*_8_, are regression coefficients for the eight non-mosquito factors, and *γ*_1_,…,*γ*_8_, are regression coefficients for the eight mosquito species. The notation 1_*i*_(*s*),1 ≤ *i* ≤ 8, are zero-one indicators: 1_*i*_(*s*) is taken as unity if the *i*th mosquito type is present in county *s* and zero otherwise.

To estimate the parameters *β*_0_, **β**_*i*_,1 ≤ *i* ≤ 8, and **γ**_*i*_,1 ≤ *i* ≤ 8, from the data, the classical method of maximum likelihood [23] is used. Once the logistic regression parameters are estimated, an estimate of *p*(*s*) based upon the fitted model is computed via

(4) $${\hat{p}}_{\text{Logistic}}(s)=\frac{e^{ĝ(X(s))}}{1+e^{ĝ(X(s))}},$$

where quantities in (4) are estimated by

(5) $$ĝ(X(s))={\hat{\beta }}_{0}+\overset{8}{\underset{i=1}{\sum }}{\hat{\beta }}_{i}f_{i}(s)+\overset{8}{\underset{i=1}{\sum }}{\hat{\gamma }}_{i}1_{i}(s).$$

## Results and discussion

**Figure 19:**
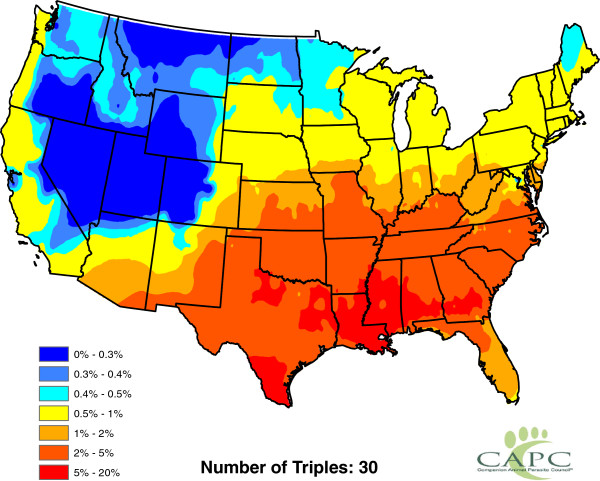
Predicted heartworm prevalence from all factors.

Figure 19 shows the results from a fitted logistic regression model after smoothing the ${\hat{p}}_{\text{Logistic}}(s)$ estimates with the weighted head-banging algorithm with 30 triples. The results reproduce the rough structure of Figure 2. The overall implication is that canine heartworm seems to be reasonably quantifiable.

Clarifying details, in the head-banging procedure, the inverse of the standard deviation of ${\hat{p}}_{\text{Logistic}}(s)$ is used as the weight. For any factor, if both 2011 and 2012 data are available (such as temperature and precipitation) then the average of the observations from the two years is used in equation 5. Otherwise, the most recent measurement is used.

The individual factors and their predictive significance are worth discussing. Table 2 lists estimates of each logistic model parameter, standard errors, odds ratios, and 95*%* confidence intervals for the odds ratios. Elaborating, the standard error is the square root of the estimated variability of the regression coefficient. Smaller standard errors imply greater precision for the parameter estimate. All *p*-values for the hypothesis test that the parameter is zero are less than 0.0001. If the parameter for a factor is in truth zero, it is not a good predictor of prevalence rates. A positive/negative parameter estimator means that the corresponding factor is judged to increase/decrease heartworm prevalance as the factor increases. Since the mosquito factors are only presence/absence indicators, a positive regression coefficient estimate means that prevalence is higher when the mosquito species is present, and lower when absent. Exp (*β*) is interpreted as the odds of a dog being heartworm positive relative to the odds of the dog being negative when the corresponding predictor increases by one unit. For mosquito factors, Exp (*β*) is interpreted directly as the odds of a dog testing heartworm positive when the corresponding mosquito species is present relative to the odds of the dog testing negative when this mosquito species is absent. Hence, if Exp (*β*) is larger than unity, then heartworm prevalence is increased by the corresponding factor, and vice versa. The units for the elevation regression coefficient estimator are in thousands of feet; the estimated coefficient for median household income is in thousands of dollars.

**Table 2:** Significance of factors**

**Effect**	**Estimate**	**Standard error**	**Exp(*β*)**	**95% Wald CI**
Intercept	-8.0610	0.0819		
Temperature	0.0720	0.0011	1.075	(1.072, 1.077)
Median household income	-0.0227	0.0003	0.978	(0.977, 0.978)
Population density	-0.0175	0.0004	0.983	(0.982, 0.984)
Precipitation	0.0982	0.0049	1.103	(1.093, 1.114)
Elevation	-0.0605	0.0030	0.941	(0.936, 0.947)
Relative humidity	-0.0147	0.0008	0.985	(0.984, 0.987)
Forest coverage	1.2493	0.1156	3.448	(2.781, 4.375)
Surface water coverage	0.1054	0.0265	1.111	(1.055, 1.170)
*Aedes trivittatus*	0.9987	0.0153	2.715	(2.634, 2.797)
*Culex quinquefasciatus*	0.4063	0.0152	1.501	(1.457, 1.547)
*Aedes sierrensis*	0.6947	0.0293	2.003	(1.892, 2.121)
*Anopheles punctipennis*	0.4132	0.0182	1.512	(1.459, 1.567)
*Anopheles quadrimaculatus*	-0.1663	0.0185	0.847	(0.817, 0.878)
*Aedes aegypti*	-0.1232	0.0112	0.884	(0.865, 0.904)
*Aedes canadensis*	0.1481	0.0143	1.160	(1.128, 1.192)
*Aedes albopictus*	-0.0894	0.0112	0.915	(0.895, 0.935)

^**^All factors are significant at level 0.01.

The model results in a fairly good fit: McFadden’s pseudo *R*^2^ = 0.62 [24]. All predictors were significant predictors of heartworm prevalence rates at level 0.0001. Higher temperatures are associated with higher prevalence – heartworm is generally more prevalent in warmer temperatures. Higher median household incomes are associated with lower prevalence rates. Logically, higher income pet owners can more easily afford heartworm preventives. Not surprisingly, heartworm prevalence decreases with increasing population density and elevation. It is surprising to see that prevalence declines with higher humidities. One explanation for this may lie with the high correlations among elevation, relative humidity, and temperature. This will be explored further below when a model that allows the factors to interact is examined.

Presence of *Culex quinquefasciatus*, *Aedes sierrensis*, *Anopheles punctipennis*, *Anopheles quadrimaculatus*, and *Aedes canadensis* are associated with higher heartworm prevalence. The other three mosquito species are estimated to decrease prevalence rates. The reader should not *a priori* believe that presence of any mosquito species acts to increase prevalences in the model: the 16 factors are being "judged" in tandem.

It is perhaps remarkable that every factor considered is judged to influence prevalence rates with a significance level of 0.0001. It leaves an unsettling feeling that other important factors may have been omitted. We reiterate that this study is exploratory and will hopefully be improved in the future. We should also mention the usual regression caveat: (3) presupposes a linear relationship on the factors that may not be realistic at all factor levels. This drawback is not germane to this study; linear regression models, of course, serve as rudimentary guidance.

The above results, often called a main effects analysis, can be improved by adding interaction terms into the regression. An interaction term between factors *i* and *j* adds an additional regression term of form *ξ*_*i*,*j*_*f*_*i*_(*s*) *f*_*j*_(*s*) into equation 3. Table 3 reports a logistic regression model fit with most non-mosquito factors allowed to pairwise interact. The notation Temperature*Elevation, for example, refers to temperature and elevation interaction. There are 82=28 possible interacting pairs. However, only 16 interaction pairs were considered due to practical constraints. For example, interaction between elevation and temperature is plausible since heartworm prevalence at higher elevations may differ according to temperature. However, median household income and temperature could not be sensibly allowed to interact since heartworm prevalence for dog owners with high salaries does not depend on the temperature where he/she lives, and vice-versa. The mosquito factors were not allowed to interact with other factors because they are simply presence/absence variables. All insignificant factors and interactions were eliminated at the 5% level with a standard backward elimination regression procedure [25]. Clarifying, we first fitted a model with all individual factors and 16 interactions. The term with the largest *p*-value in the regression was eliminated if its *p*-value exceeded 0.05 and the model was refitted. This procedure was repeated until all insignificant factors were eliminated at the 5% level.

**Table 3:** Significance factors and interactions*

**Effect**	**Estimate**	**Standard error**
Intercept	-8.4312	0.0993
Median household income	-0.0267	0.0004
Temperature	0.0655	0.0015
Elevation	0.5609	0.0171
Population density	-0.0444	0.0015
Temperature*Elevation	-0.0087	0.0003
Temperature*Forest coverage	0.4840	0.0195
Temperature*Surface water coverage	-0.0345	0.0034
Elevation*Relative humidity	-0.0041	0.0002
Forest coverage	-36.5371	2.1127
Population density*Median household	0.0005	<0.0001
income		
Forest coverage*Surface water coverage	-18.1276	1.5896
Precipitation	0.3049	0.0278
Precipitation*Surface water coverage	-0.3247	0.0299
Precipitation*Elevation	0.0196	0.0020
Precipitation*Relative humidity	-0.0032	0.0004
Elevation*Surface water coverage	0.2472	0.0342
Elevation*Woodland coverage	0.7325	0.1019
Forest coverage*Relative humidity	0.0903	0.0234
Surface water coverage*Relative humidity	0.0281	0.0075
Surface water coverage	1.1773	0.5507
*Aedes trivittatus*	1.0682	0.0158
*Aedes sierrensis*	1.1311	0.0313
*Culex quinquefasciatus*	0.5483	0.0163
*Anopheles punctipennis*	0.3948	0.0191
*Aedes canadensis*	0.1567	0.0151
*Aedes albopictus*	-0.0976	0.0116
*Aedes aegypti*	-0.0879	0.0126
*Anopheles quadrimaculatus*	-0.1084	0.0163

^*^All factors and their interactions are significant at level 0.01, except for that surface water coverage is significant at level 0.05.

Including interactions increased McFadden‘s pseudo *R*^2^ to 0.65. Table 3 summarizes the results of this procedure. In this table, Exp(*β*) and their confidence intervals were not included. This is because when factor interactions are included, the value of Exp(*β*) depends not only on the coefficient of this factor, but also on all the other factors that interact with the factor. All listed factors are significant with a significance level of 0.0001, except the surface water coverage*relative humidity interaction (*p*-value of 0.0002) and surface water coverage (*p*-value of 0.0327). Differences from the Table 2 results exist, but are not radical. Relative humidity, for example, is not itself significant but interacts with several other factors. All other individual factors remain significant predictors. The parameter estimates of the main effects are slightly different from those in Table 2 since interactions are now included. For example, the parameter estimates of both median household income and population density are smaller than those in Table 2 and their interaction is significant. The significant interaction indicates that the changes of heartworm prevalence as median household income changes are conditional on the value of population density, and vice versa.

**Figure 20:**
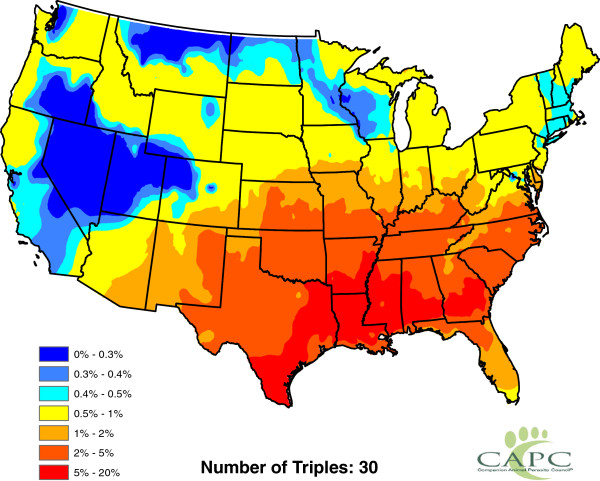
Predicted heartworm prevalence from significant factors and interactions.

Figure 20 presents an analogous graphic to Figure 19 when interactions were included in the logistic regression model and predictions are made from the fitted model in Table 3. Results allow for interactions and a backward selection was used to eliminate any insignificant factors and their interactions. The results are comparable to those in Figure 19.

## Conclusions

Our factor list in Table 1 is likely incomplete; however, it should serve as a starting point. Several important factors that were discussed in Brown *et al.*[2] could not be included in the analysis due to data deficiencies. For example, coyotes and feral dogs are known reservoirs of heartworm infection. Unfortunately, no complete data of their populations were available. Additionally, mosquito vector abundance data were unavailable. Should information regarding coyote counts and vector abundance be consistently collected in the future and incorporated in the methods discussed herein, the model would improve in power and practical implications.

Some of the factors could be refined. For example, it is frequently posited that heartworm degree units for temperature likely influence prevalence rates [26]. Such units are typically measured from 14 degree Celsius (57 degrees Fahrenheit), below which it is difficult to have high heartworm transmission. We have not looked at temperature departures above 14 degree Celsius because our data are annual. Also, subtracting 14 from the annual temperatures would reparametrize the value of *β*_0_, but would not change the overall regression fit.

The fitted regression model is currently being explored to forecast future values of heartworm prevalence. Indeed, many of the predictive factors vary with time (temperature and precipitation, for example). From a forecast of these factors — say a year in advance — and our fitted logistic regression model, predictions of prevalence rates can be made. This can inform the pet owner and practitioner in advance of a potentially bad heartworm season. The results can also be used to assess prevalence rate changes due to climate change. For example, if annual temperatures are expected to increase by one degree F, one could add one degree to the temperature in the fitted logistic regression model to predict the change.

## Competing interests

The authors have no competing interests relative to the work presented in this report.

## Authors’ contributions

All authors were responsible for identifying candidate factors. DW, JS, and RL were responsible for the statistical analysis and the production of figures and tables. RL and DDB wrote the draft document. All authors revised the paper and agreed with its final version.

## References

[B1] CAPC parasite prevalence maps heartworm [ http://www.capcvet.org/parasite-prevalence-maps]

[B2] Brown HE Harrington LC Kaufman PE McKay T Bowman DD Nelson CT Wang D Lund R Key factors influencing canine heartworm, Dirofilaria immitis, in the United States Parasites Vectors 2012 5 245 10.1186/1756-3305-5-245 23111089 PMC3523980

[B3] Sacks BN Woodward DL Colwell AE A long-term study of non-native-heartworm transmission among coyotes in a Mediterranean ecosystem Oikos 2003 102 478 490 10.1034/j.1600-0706.2003.12590.x

[B4] Levy JK Lappin MR Glaser AL Birkenheuer AJ Anderson TC Edinboro CH Prevalence of infectious disease in cats and dogs rescued following Hurricane Katrina J Am Vet Med Assoc 2011 238 311 317 10.2460/javma.238.3.311 21281213

[B5] ANTECH Diagnostics [ http://www.antechdiagnostics.com/Main/Home.aspx]

[B6] IDEXX Laboratories [ http://www.idexx.com/view/xhtml/en_us/corporate/home.jsf]

[B7] National climatic data center [ http://www7.ncdc.noaa.gov/CDO/cdoselect.cmd?datasetabbv=GSOD&countryabbv=&georegionabbv]

[B8] National climatic data center [ http://www.ncdc.noaa.gov/monitoring-references/maps/us-climate-divisions.php]

[B9] Paroscientific, Inc [ http://www.paroscientific.com/dewpoint.htm]

[B10] Peakbagger.com [ http://peakbagger.com]

[B11] United States department of agriculture [ http://quickstats.nass.usda.gov/]

[B12] Census bureau [ http://www.census.gov/support/USACdataDownloads.html\#LND]

[B13] Census bureau [ http://factfinder2.census.gov/faces/nav/jsf/pages/searchresults.xhtml?refresh=t]

[B14] Bureau of labor statistics, U.S. Department of labor [ http://www.bls.gov/cpi]

[B15] Moore CG Exotic mosquitoes in the USA: updates and trends27th Biennial State Public Health Vector Control Conference March 25–26, 2008 Fort Collins, Colorado [ http://www.cdc.gov/ncidod/dvbid/westnile/conf/27thbiennialVectorControl/index.htm]

[B16] Hynes NA Dengue: a reemerging concern for travelers Clevel Clin J Med 2012 79 7 474 482 10.3949/ccjm.79a.11048 22751631

[B17] Darsie RF Jr Ward RA Identification and Geographical Distribution of the Mosquitoes of North America, North of Mexico 2004:384 Gainseville: University Press of Florida

[B18] Wilson EB Probable inference, the law of succession, and statistical inference J Am Stat Assoc 1927 22 209 212 10.1080/01621459.1927.10502953

[B19] Tukey PA Tukey JW Graphical Display of Data Sets in 3 or More Dimensions, Interpreting Multivariate Data 1981 New York: Wiley

[B20] Mungiole M Pickle LW Simonson KH Application of a weighted head-banging algorithm to mortality data maps Stat Med 1999 18 3201 3209 10.1002/(SICI)1097-0258(19991215)18:23<3201::AID-SIM310>3.0.CO;2-U 10602145

[B21] Hansen KM Head-banging: robust smoothing in the plane IEEE Trans Geosci Remote Sensing 1991 29 369 378 10.1109/36.79427

[B22] Pohlman JT Leitner DW A comparison of ordinary least squares and logistic regression Ohio J Sci 2003 103 n5 118 125

[B23] Czepiel SA Maximum likelihood estimation of logistic regression models: theory and implementation [ http://czep.net/contact.html]

[B24] McFadden D Zarembka P Conditional logit analysis of qualitative choice behavior Frontiers In Econometrics 1973 New York: Academic Press 105 142

[B25] Hosmer DW Lemeshow S Model-building strategies and methods for logistic regression Applied Logistic Regression, 2nd Edition 2000 New Jersey: John Wiley & Sons, Inc.

[B26] Shearer P Literature review - heartworm disease Banfield Appl Res & Knowl Team 2011 1 16 [ http://www.banfield.com/getmedia/e456eec3-77f2-46f1-b302-87a17a8fba0a/5906a327-4e82-49f7-9432-e7da7fc568a9-pdf0]

